# Cardiomyopathies in Children and Systemic Disorders When Is It Useful to Look beyond the Heart?

**DOI:** 10.3390/jcdd9020047

**Published:** 2022-01-31

**Authors:** Valentina Lodato, Giovanni Parlapiano, Federica Calì, Massimo Stefano Silvetti, Rachele Adorisio, Michela Armando, May El Hachem, Antonino Romanzo, Carlo Dionisi-Vici, Maria Cristina Digilio, Antonio Novelli, Fabrizio Drago, Massimiliano Raponi, Anwar Baban

**Affiliations:** 1 The European Reference Network for Rare, Low Prevalence and Complex Diseases of the Heart-ERN GUARD-Heart, Pediatric Cardiology and Arrhythmia/Syncope Units, Bambino Gesù Children Hospital and Research Institute, IRCCS, 00165 Rome, Italy; valentina.lodato@opbg.net (V.L.); giovanni.parlapiano@opbg.net (G.P.); federica.cali@opbg.net (F.C.); mstefano.silvetti@opbg.net (M.S.S.); fabrizio.drago@opbg.net (F.D.); 2 Laboratory of Medical Genetics, Bambino Gesù Children Hospital and Research Institute, IRCCS, 00165 Rome, Italy; antonio.novelli@opbg.net; 3 Heart Failure Clinic-Heart Failure, Heart Transplant, Mechanical Circulatory Support Unit, Department of Pediatric Cardiology and Cardiac Surgery, Heart and Lung Transplant, Bambino Gesù Children Hospital and Research Institute, IRCCS, 00165 Rome, Italy; rachele.adorisio@opbg.net; 4 Department of Neuroscience and Neurorehabilitation, Bambino Gesù Children Hospital and Research Institute, IRCCS, 00165 Rome, Italy; michela.armando@opbg.net; 5 Dermatology and Genodermatosis Units, Genetics and Rare Disease Research Division, Bambino Gesù Children Hospital and Research Institute, IRCCS, 00165 Rome, Italy; may.elhachem@opbg.net; 6 Ophtalmology Unit, Bambino Gesù Children Hospital and Research Institute, IRCCS, 00165 Rome, Italy; antonino.romanzo@opbg.net; 7 Division of Metabolism, Bambino Gesù Children Hospital and Research Institute, IRCCS, 00165 Rome, Italy; carlo.dionisivici@opbg.net; 8 Genetics and Rare Diseases Research Division, Bambino Gesù Children Hospital and Research Institute, IRCCS, 00165 Rome, Italy; mcristina.digilio@opbg.net; 9 Medical Direction, Bambino Gesù Children Hospital, IRCCS, 00165 Rome, Italy; massimiliano.raponi@opbg.net

**Keywords:** cardiomyopathies, children, syndromes, heterogeneity, multisystemic, personalized approach

## Abstract

Cardiomyopathy (CMP) is a rare disease in the pediatric population, with a high risk of morbidity and mortality. The genetic etiology of CMPs in children is extremely heterogenous. These two factors play a major role in the difficulties of establishing standard diagnostic and therapeutic protocols. Isolated CMP in children is a frequent finding, mainly caused by sarcomeric gene variants with a detection rate that can reach up to 50% of analyzed cohorts. Complex multisystemic forms of pediatric CMP are even more heterogenous. Few studies in literature take into consideration this topic as the main core since it represents a rarity (systemic CMP) within a rarity (pediatric population CMP). Identifying etiology in this cohort is essential for understanding prognosis, risk stratification, eligibility to heart transplantation and/or mechanical-assisted procedures, preventing multiorgan complications, and relatives’ recurrence risk calculation. The previous points represent a cornerstone in patients’ empowerment and personalized medical care approach. The aim of this work is to propose a new approach for an algorithm in the setting of the diagnostic framework of systemic pediatric CMP. On the other hand, during the literature review, we noticed a relatively common etiologic pattern in some forms of complex/multisystem CMP. In other words, certain syndromes such as Danon, Vici, Alström, Barth, and Myhre syndrome share a common pathway of directly or indirectly defective “autophagy” process, which appears to be a possible initiating/triggering factor for CMPs. This conjoint aspect could be important for possible prognostic/therapeutic implications in this category of patients. However, multicentric studies detailed functional and experimental models are needed prior to deriving conclusions.

## 1. Introduction

### Insight in Pathogenesis

Cardiomyopathy (CMP) is a rare disease in childhood with a high risk of morbidity and mortality [1,2]. CMP is the primary indication for heart transplantation (HT) in children, particularly >one year of age [2,3,4,5].

Previous population studies estimate the incidence of primary CMPs in children to be 1:100,000 in individuals <20 years old [1,2,6]. It seems to be generally higher in male individuals, Africans, and aboriginal Australian children [1,2]. CMP in children can be classified into dilated cardiomyopathy (DCM), which represents 50% of patients (of whom 10–25% are secondary to acute myocarditis); hypertrophic cardiomyopathy (HCM) in 35–50%; restrictive cardiomyopathy (RCM) in <5% of CMP in children; left ventricular (LV) non-compaction (LVNC) in ≈5% of CMP; while no specific data are available regarding the incidence of arrhythmogenic cardiomyopathy (ACM) in children’s populations [1,2,6,7,8,9].

Pediatric CMPs are highly heterogenous in origin. It ranges from genetics to acquired causes. Genetically determined pediatric CMPs can be isolated (merely cardiac involvement) or systemic (multiorgan involvement) [10].

Isolated CMPs in children are mainly autosomal-dominant (AD) [1]. However, autosomal-recessive (AR) and X-linked forms are rarely observed [10]. There are tens of genes that are known to be majorly involved in CMPs. Candidate genes can reach hundreds, and the di-/multigenic origin of CMPs in children is a real challenge for physicians. Double or triple genetic hits in early or severe onset CMP is being increasingly observed in studies. Pathogenic variants in sarcomeric genes are the most common abnormalities in children with isolated HCM, RCM, LVNC, and DCM [11,12,13,14,15,16]. Desmosomal gene variants are associated with ACM. Other studies reflect the light of potential predisposition to ACM secondary to channelopathies [9,17].

The genetics of CMPs in children is extremely heterogenous, complex, potentially underestimated, and underdiagnosed mainly due to rarity and difficulties in establishing homogenous protocol studies in this specific cohort [18].

Complex multisystemic/multiorgan/syndromic forms of pediatric CMP are even more heterogenous and challenging. Few studies in literature take into consideration this topic as the main core since it represents a rarity (systemic CMP) within a rarity (pediatric population CMP).

The identification of etiologies in systemic disorders in pediatric CMP can be important for understanding prognosis, risk stratification, eligibility to HT and/or mechanical assist procedures, preventing multiorgan complications, and recurrence risk calculation of relatives. The previous points represent a cornerstone in patients’ empowerment and personalized medical care approach.

**Figure 1:**
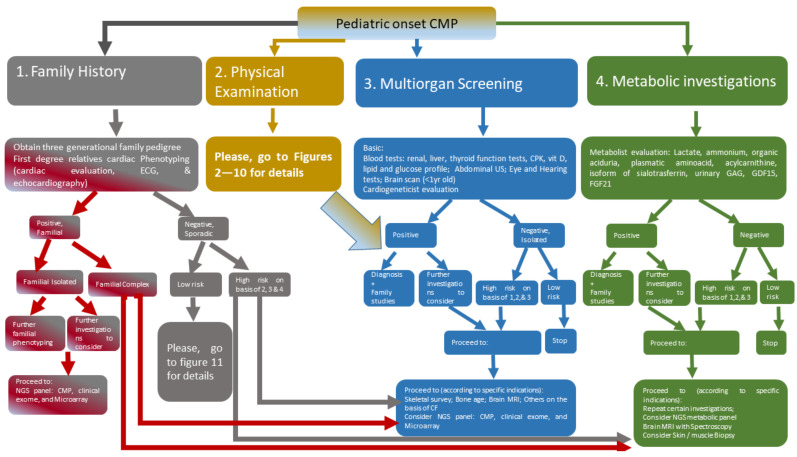
Diagnostic algorithm in pediatric-onset CMP. The algorithm brings light to a step-by-step method for the clinical approach when facing the management of a cohort with CMP in children. Abbreviations: CF, clinical features; CMP, cardiomyopathy; CPK; creatine phosphokinase; ECG electrocardiography; FGF21, fibroblast growth factor 21; GAG, glycosaminoglycan; MRI, magnetic resonance imaging; GDF15, growth differentiation factor 15; NGS, next-generation sequencing; US, ultrasound.

The aim of this work is to propose a systematic approach for an algorithm in the setting of the diagnostic framework of systemic pediatric CMP (Figure 1). In other words, when a physician faces a child with CMP (the tip of the iceberg) in a potential systemic context (the rest of the iceberg), what are the major steps to follow in order to reveal the remaining components of that specific condition? Our method is based on what major studies show in literature and what we have identified on the basis of our observed cohort in the last ten years of 600 children with CMP.

In this systemic revision of literature, we opted to bring light to certain rare disorders of different etiological backgrounds. Within the metabolic conditions, we are aware of not reporting certain major groups, such as mucopolysaccharidosis, due to its extensive description in studies. We avoided repeating common examples and focused on these lesser-known disorders or those with expanding phenotypic spectrum within the last few years.

## 2. Deciphering Etiology of CMPs in Children according to Major Multisystemic Clinical Features

**Figure 2:**
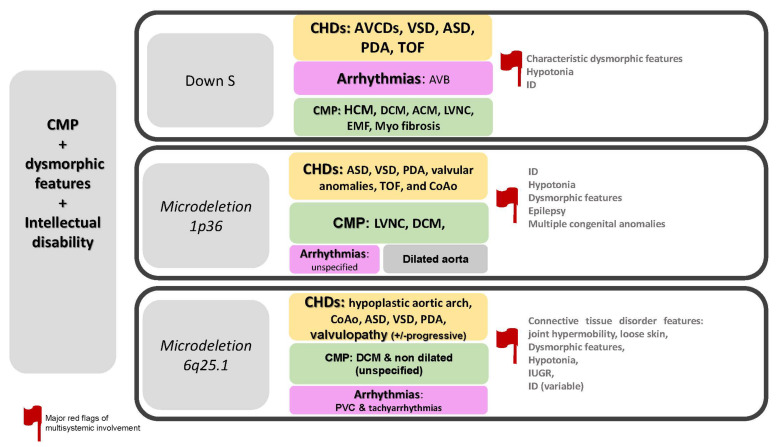
CMP + dysmorphic features + intellectual disability. Abbreviations: ACM; arrhythmogenic cardiomyopathy; ASD, atrial septal defects; AVB, atrioventricular block; AVCDs, atrioventricular canal defects; CHDs, congenital heart defects; CMP, cardiomyopathy; CoAo, coarctation of aorta; DCM, dilated cardiomyopathy; EMF, endomyocardial fibrosis; ID, intellectual disability; IUGR, intrauterine growth restriction; HCM; hypertrophic cardiomyopathy; LVNC, left ventricular non-compaction; PDA, patent ductus arteriosus; PVC, premature ventricular contraction; S, syndrome; TOF, tetralogy of Fallot; VSD, ventricular septal defect.

**Figure 3:**
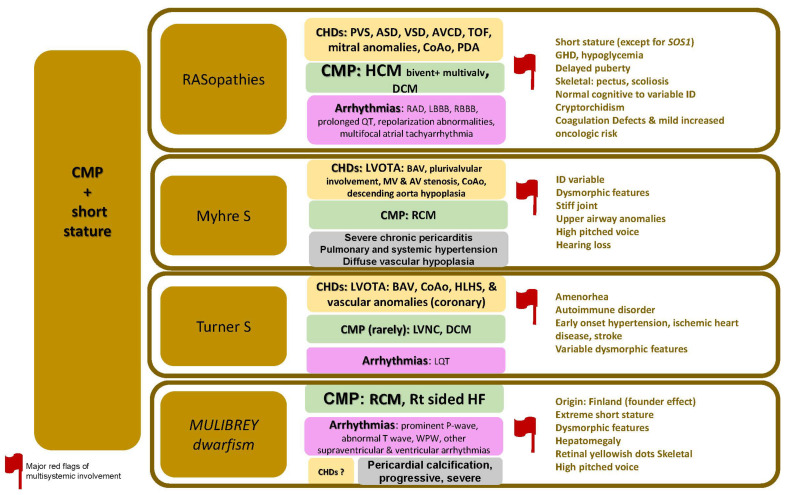
CMP + short stature. Abbreviations; ASD, atrial septal defects; AV, aortic valve; AVCDs, atrioventricular canal defects; BAV, bicuspid aortic valve; CHDs, congenital heart defects; CMP, cardiomyopathy; CoAo, coarctation of aorta; DCM, dilated cardiomyopathy; ID, intellectual disability; GHD, growth hormone deficiency; HCM; hypertrophic cardiomyopathy; HF, heart failure; HLHS, hypoplastic left heart syndrome; LBBB, left bundle branch block; LQT, long QT; LVNC, left ventricular non-compaction; PDA, patent ductus arteriosus; LVOTA, left ventricular outflow tract area; MV, mitral valve; PVS, pulmonary vein stenosis; RAD, right axis deviation; RBBB, right bundle branch block; RCM, restrictive cardiomyopathy; S, syndrome; Rt, right side; TOF, tetralogy of Fallot; VSD, ventricular septal defect; WPW, Wolff Parkinson White.

**Figure 4:**
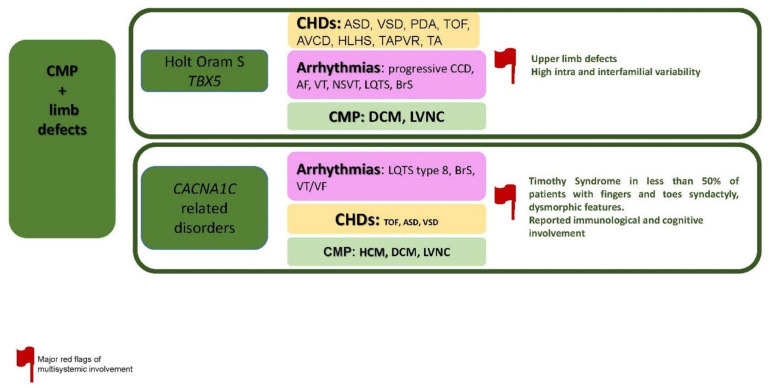
CMP + limb defects. Abbreviations: AF, atrial fibrillation; ASD, atrial septal defects; AVCD, atrioventricular canal defects; BrS, Brugada Syndrome; CCD, cardiac conduction defects; CHDs, congenital heart defects; CMP, cardiomyopathy; DCM, dilated cardiomyopathy; HCM; hypertrophic cardiomyopathy; HLHS, hypoplastic left heart syndrome; LQTS, long QT syndrome; LVNC, left ventricular non-compaction; NSVT, non-sustained ventricular tachycardia; PDA, patent ductus arteriosus; S, syndrome; TA, troncus arteriosus; TOF, tetralogy of Fallot; TAPVR, total anomalous pulmonary venous return; VF, ventricular fibrillation; VSD, ventricular septal defect; VT, ventricular tachycardia.

**Figure 5:**
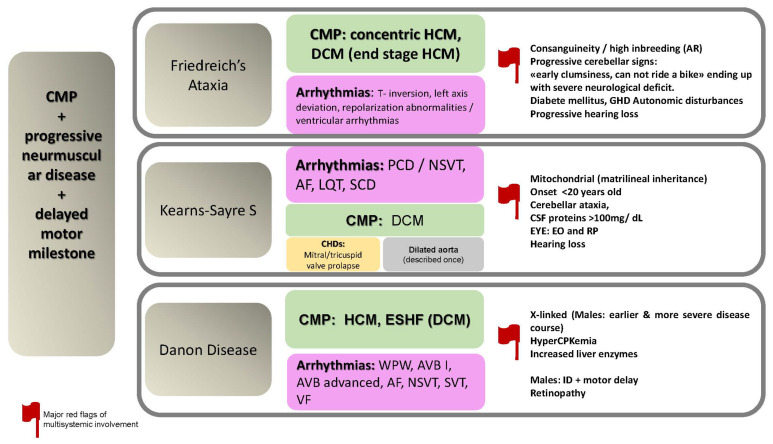
CMP + progressive neuromuscular disease + delayed motor milestone. Abbreviations: AF, atrial fibrillation; AR, autosomal recessive; AVB, atrioventricular block; CHDs, congenital heart defects; CMP, cardiomyopathy; CSF, cerebrospinal fluid; DCM, dilated cardiomyopathy; CPK; creatine phosphokinase; EO, external ophthalmoplegia; ESHF, end-stage heart failure; GHD, growth hormone deficiency; HCM; hypertrophic cardiomyopathy; ID, intellectual disability; LQT, long QT; NSVT, non-sustained ventricular tachycardia; PCD, progressive conduction defects; RP, retinitis pigmentosa; S, syndrome; SCD, sudden cardiac death; SVT, sustained ventricular tachycardia; VF, ventricular fibrillation; WPW, Wolff Parkinson White.

**Figure 6:**
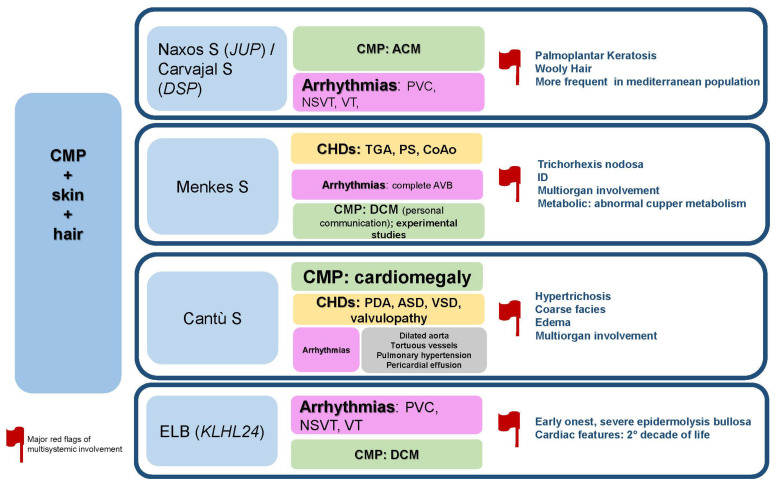
CMP + skin + hair. Abbreviations: ACM; arrhythmogenic cardiomyopathy; ASD, atrial septal defects; AVB, atrioventricular block; CHDs, congenital heart defects; CMP, cardiomyopathy; CoAo, coarctation of aorta; DCM, dilated cardiomyopathy; ID, intellectual disability; NSVT, non-sustained ventricular tachycardia; PDA, patent ductus arteriosus; PVC, premature ventricular contraction; PS, pulmonary stenosis; S, syndrome; TGA, transposition of great arteries, VSD, ventricular septal defect; VT, ventricular tachycardia.

**Figure 7:**
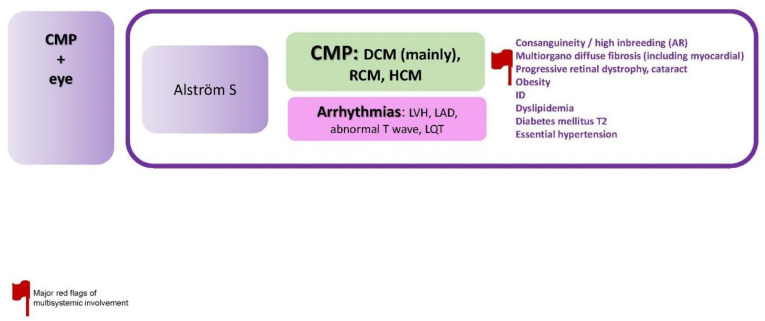
CMP + eye. Abbreviations: AR, autosomal recessive; CMP, cardiomyopathy; DCM, dilated cardiomyopathy; HCM; hypertrophic cardiomyopathy; ID, intellectual disability; LAD, left axis deviation; LQT, long QT; LVH, left ventricular hypertrophy; RCM, restrictive cardiomyopathy; S, syndrome; T2, type 2.

**Figure 8:**
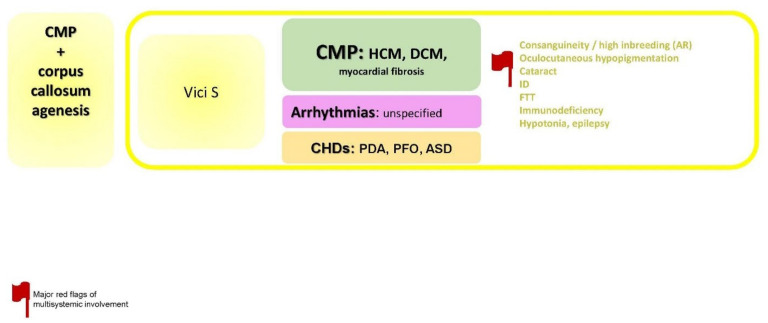
CMP + corpus callosum agenesis. Abbreviations: ASD, atrial septal defects; AR, autosomal recessive; CHDs, congenital heart defects; CMP, cardiomyopathy; DCM, dilated cardiomyopathy; FTT, failure to thrive; HCM; hypertrophic cardiomyopathy; ID, intellectual disability; PDA, patent ductus arteriosus; PFO, patent forame ovale.

**Figure 9:**
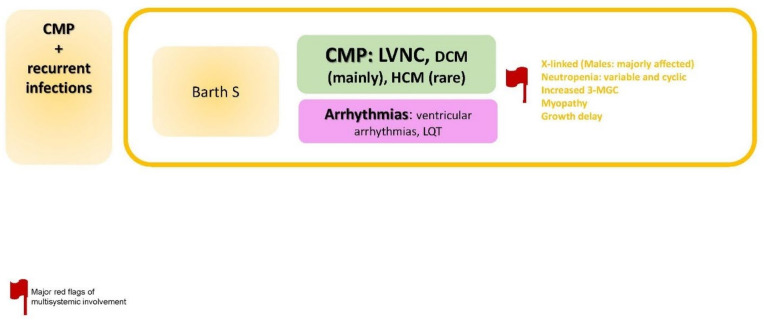
CMP + recurrent infections. Abbreviations: 3-MGC, methylglutaconic aciduria; CMP, cardiomyopathy; DCM, dilated cardiomyopathy; HCM; hypertrophic cardiomyopathy; LQT, long QT, LVNC, left ventricular non-compaction; S, syndrome.

**Figure 10:**
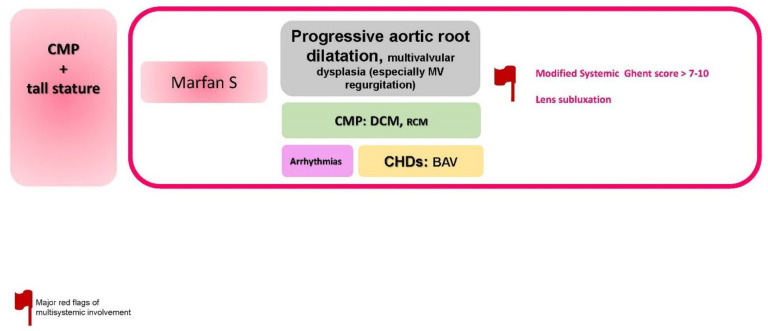
CMP + tall stature. Abbreviations: BAV, bicuspid aortic valve; CMP, cardiomyopathy; CHDs, congenital heart defects; DCM, dilated cardiomyopathy; MV, mitral valve, RCM, restrictive cardiomyopathy; S, syndrome.

In this section, we describe major genetic conditions that are related to pediatric CMP in a multisystemic/complex/syndromic context. We classified these disorders into main subgroups in accordance with the major extracardiac clinical findings that are often associated with CMP. Systemic and cardiac red flags for specific disorders are highlighted to broaden clinicians’ knowledge for rapid and early framing towards prompt and appropriate diagnosis and multidisciplinary management. These data are reported both in the following paragraphs and in the specific figure for each delineated disorder (Figure 2, Figure 3, Figure 4, Figure 5, Figure 6, Figure 7, Figure 8, Figure 9 and Figure 10).

### 2.1. CMP + Dysmorphic Features + Intellectual Disability

The specific disorders reported in this paragraph is summarized in Figure 2.

#### 2.1.1. Down Syndrome (DS)

Down syndrome (DS, MIM # 190685) is the most common chromosomal aneuploidy syndrome with a prevalence of 1–5/10,000.

DS is commonly characterized by short stature, muscle hypotonia, atlantoaxial instability, reduced neuronal density, cerebellar hypoplasia, intellectual disability (ID), congenital heart defects (CHDs) (44–58%) and possible development of hypothyroidism, autoimmune diseases, obstructive sleep apnoea, epilepsy, hearing and vision problems, haematological disorders (including leukaemia), recurrent infections, anxiety disorders and early-onset Alzheimer’s disease [18,19,20].

CMP in DS is rarely reported in studies, but primary myocardial involvement in DS is previously reported, including HCM, ACM, endomyocardial fibroelastosis, LVNC, myocardial fibrosis and unspecified CMP [20,21,22,23].

Previous studies in literature had revealed preliminary results of a higher incidence of biventricular dysfunction in patients with DS, even in the absence of CHDs compared with healthy controls. These findings suggest the importance of serial screening with cardiac evaluation, electrocardiogram (ECG) and conventional echocardiography in this specific cohort [24,25].

#### 2.1.2. 1p36 Deletion Syndrome

Chromosome 1p36 deletion syndrome (MIM # 607872) is a copy number variant (CNV) with an incidence rate of 1:5000–10,000. It consists of a contiguous gene syndrome characterized mainly by congenital anomalies and ID [26,27,28].

The chromosomal-deleted region ranges from 1.5 Mb to 10.5 Mb, and two critical regions are known: the distal critical region and proximal critical region [29]. Terminal deletions of chromosome 1p36 are related to the high risk of cardiac-related abnormalities [30].

The majority of patients with 1p36 deletion syndrome show typical craniofacial dysmorphisms, developmental delay (in up to 90%) and multiple congenital anomalies: malformations of the central nervous system (CNS) (88%), seizures (44–79%), heart defects (71–75%), skeletal anomalies (41%), vision problems, hearing loss, and other less frequent features. The phenotypic variability might be related to the involvement of specific genes harbored in the deleted region [31].

1p36 deletion is associated with cardiovascular abnormalities, which can be subdivided into CHDs, CMPs and potential progressive aortic dilatation [30,31,32].

The most common CHDs described are atrial and ventricular septal defects (ASD, VSD), patent ductus arteriosus (PDA), valvular anomalies, tetralogy of Fallot (TOF) and coarctation of the aorta (CoAo) [30].

Data from literature estimates a range of CMP from 23% to 31% [33,34,35,36]. The majority are LVNC and less frequently severe DCM [30,31,32,33,34,35,36].

In an observational study in our center, we described LVNC in 55% (5/9) of patients, with normal LV function in one, mildly reduced LV function in three, and severe LV dysfunction in one patient [32]. These data show wide variability in myocardial involvement in this specific disorder.

#### 2.1.3. 6q25.1 Deletion Syndrome

6q25.1 deletion syndrome (MIM # 612863) is another CNV recurrent deletion syndrome, which is recently attentional for its cardiac involvement. 6q25.1 chromosomal region harbors *TAB2* (OMIM # 605101), whose haploinsufficiency is characterized by CHDs, CMP and other minor anomalies [18,37].

Extracardiac phenotypic spectrum related to 6q25.1 deletion syndrome and *TAB2* pathogenic variants is highly variable, including facial dysmorphisms, intrauterine growth restriction (IUGR), short stature, hypotonia, and connective tissue abnormalities such as joint laxity or hypermobility, developmental delay and possible ID [37].

Connective tissue abnormalities are frequently reported, including joint hypermobility, umbilical and inguinal hernias, skeletal and/or skin abnormalities and hypotonia [38].

CHD is observed in 71% of patients with 6q25.1 deletion with high intra- and interfamilial variability ranging from ventricular outflow tract lesions including a bicuspid aortic valve (BAV), hypoplastic aortic arch, CoAo, pulmonary valve dysplasia, ASDs, VSDs, aortic dilation, mitral and tricuspid valve abnormalities. The arrhythmic spectrum varies from premature ventricular contractions (PVC) to tachyarrhythmias [18,37,39,40,41].

Early age CMP is described in 6q25.1 deletion syndrome (34%) with significant myocardial dysfunction not CHD related [18,37,38]. In a similar manner, *TAB2* pathogenic variants seem to cause CMP with biventricular failure [42].

These data need further observation before reaching definitive conclusions regarding the percentage of clinical characteristics due to the rarity of the condition.

### 2.2. CMP + Short Stature 

The specific disorders reported in this paragraph is summarized in Figure 3.

#### 2.2.1. RASopathies

RASopathies are a group of disorders caused by pathogenic variants in a group of genes within the signal transducers of the RAS-MAPK cascade. It is relatively frequent and affects 1 in 1000–2500 children [43,44]. Mild expression is likely to be overlooked. Among these disorders, Noonan syndrome (NS, MIM # 163950), NS with multiple lentigines (NSML, MIM # 151100, also known with the acronym LEOPARD syndrome), Costello syndrome (CS, MIM # 218040) and cardiofaciocutaneus syndrome (CFCS, MIM # 115150). It is caused by more than 15 genes, including (*PTPN11, RAF1, SOS1, SOS2, BRAF, NRAS, KRAS, HRAS, MRAS, RRAS2, RIT1, LZTR1, MAP2K1, MAP2K2, CBL, SHOC2* and *SPRED2*). Many affected individuals have de novo variants; however, an affected parent is recognized in 30–75% of families.

Cardiovascular manifestations are very frequent and reach 85 to 90% of patients. Variable CHDs are described: pulmonary valve stenosis (25–71%) with doming supravalvular component in the majority of cases, ASD (4–57%), VSD (1–14%), atrio-ventricular canal defect (AVCD) (1–13%), mitral abnormalities 2–17%, CoAo 2–9%, PDA 1–6% and TOF 1–4% [45,46,47].

Regarding myocardial abnormality, the predominant form is HCM which reaches 10–29% of RASopathies. On the other hand, according to a large clinical registry, RASopathies may represent the underlying diagnosis in 18% of childhood HCM, and it accounts for 42% of infants with HCM [48,49,50,51]. The major red flags associated with HCM in RASopathy often include biventricular hypertrophy (*PTPN11*), plurivalvular abnormalities: majorly pulmonary (*PTPN11, SOS1, RAF1, RIT1, SHOC2, NRAS, CBL, BRAF1, MAP2K1, MAP2K2, KRAS, HRAS*) but not excluding mitral valve dysplasia (*RAF1, PTPN11, SOS1, RIT1, SHOC2, CBL, KRAS*). ECG abnormalities can reach up to 80% with or without major impact. They include extreme right-axis deviation (northwest axis), left or right bundle branch block (NSML), prolonged QT (NSML), repolarization abnormalities, multifocal atrial tachyarrhythmia (CS).

Extracardiac features are highly variable, and RASopathy can be overlooked when major organ involvement is spared. Characteristic facial features can evolve over time and be missed in adulthood. They include a broad forehead, down slanting palpebral fissures, hypertelorism, low set ears, pterygium colli, epicanthal folds, and a short and depressed nasal root. Growth delay is frequent in RASopathy, especially when HCM has a major hemodynamic impact. However, growth abnormality can be missing, or, on the contrary, an overgrowth can be seen in certain subgroups as in *SOS1* related RASopathy. Musculoskeletal involvement is frequent, including scoliosis and pectus deformity. Other features that can be seen are dermatologic (cafe’ -au-lait spots, acanthosis nigricans, keratosis pilaris), genitourinary (cryptorchidism), neurologic (infrequent ID), haematologic (coagulation defects, thrombocytopenia, NS/myeloproliferative disease), oncologic (juvenile myelomonocytic leukemia, lymphedema), gastrointestinal, sensorineural (hearing loss, laryngomalacia, cataract, strabismus), and endocrinologic (growth hormone deficiency, hypoglycemia, delayed puberty, and short stature) [44,52].

#### 2.2.2. Myhre Syndrome

Myhre Syndrome (MIM # 139210) is an ultra-rare monogenic AD condition characterized by multisystem involvement of connective tissue with typically decreased joint mobility, associated with possible proliferative fibrosis, ID and behavioral abnormalities [53]. The disease is known to be caused by de novo pathogenetic variants of *SMAD4* (OMIM # 600993), 18q21. *SMAD4* is a member of the TGFB1 (transforming growth factor-beta 1) signaling pathway involved in the regulation of gene expression. Several studies report *TGFB1* modulation in the process of autophagy depending on *SMAD4* status in tumoral models [54].

The condition is highly extensive in its systemic manifestation, including a distinctive short stature, characteristic facial features, nasal/high-pitched voice, hearing loss, upper airway abnormalities (choanal down laryngo-tracheal anomalies), arthropathy with stiff joints, radiographic defects, and variable degree of ID.

Cardiac involvement in Myhre syndrome is very common and highly variable (approximately 70% of cases). The most common CHD forms encompass various degrees of left-sided heart lesions such as polyvalvar dysplasia, dysplastic mitral valve stenosis, aortic valve stenosis, juxtaductal coarctation, descending aorta hypoplasia. Arterial pathology can also affect other districts in the form of narrowing, such as descending thoracic and abdominal aorta, peripheral arteries and, less commonly, peripheral pulmonary artery stenosis. These findings can lead to arterial hypertension and serious difficulties when hemodynamic studies need to be undertaken due to the risk of vascular accidents (personal observation). Other CHDs are also observed, such as ASD and VSD, and PDA [55].

The progressive cardiac spectrum includes chronic and severe pericarditis and both systemic and pulmonary hypertension.

Regarding CMP, Starr et al., in 2015, suggested in their case series a possible pathophysiological process predisposing to RCM in up to 9% of patients. The authors also point out the secondary risks to transplant surgery in Myhre patients due to an abnormal tissue response, anastomotic stenosis and deleterious myocardial remodeling [56].

#### 2.2.3. Turner Syndrome (TS)

Turner syndrome (TS) is a common cytogenetic abnormality occurring in 1 to 5 of 10,000 newborns. It is associated with a completely or partially missing X chromosome.

TS females show short statures, delayed puberty, ovarian dysgenesis, hypogonadotropic hypogonadism, infertility, CHDs, endocrine and autoimmune disorders [57].

CHDs are reported in 23% to 50% of individuals with TS, including mainly left ventricular outflow tract anomalies (LVOTA) and vascular abnormalities. Among LVOTA, BAV has a prevalence of 15–30% and CoAo of 7–18% in TS [18,58,59,60]

Rarely hypoplastic left heart syndrome (HLHS), mitral valve anomalies, congenital coronary artery anomalies and emerging new signs such as hypertension, ischemic heart disease and stroke are reported as associated with TS [18,58,59,60].

Tachycardia is more frequent in TS, and prolonged rate-corrected QT interval (QTc) has been described, but its real prevalence is actually unknown [61,62].

The association between TS and CMP is rarely reported in studies, and it includes LVNC, which in selected cases can lead to heart failure (HF) [18,63,64].

#### 2.2.4. MULIBREY Dwarfism (MUL)

MULIBREY nanism syndrome is an ultra-rare AR disease (MUL; MIM # 253250), with less than 150 patients reported since its initial description. It is caused by biallelic pathogenic variants in *TRIM37* (OMIM # 605073) on chromosome 17q22-q23 encoding a peroxisomal Ubiquitin E3 ligase. Along with profound growth delays. MULIBREY is derived from the acronym MUscle, LIver, BRain and EYe tissue changes [65].

The diagnostic major and minor criteria include the followings: growth failure in 90–95% (which can be congenital small for gestational age–SGA- and/or progressive with final height adults 3.0 standard deviation score SDS below population); characteristic radiological findings (long slender bones with thick cortex and narrow medullar channels in 93%, low and shallow J-shaped sella turcica in 89%); craniofacial abnormalities (90%) including scaphocephaly, triangular face, high and broad forehead, low nasal bridge and telecanthus, eye findings with yellowish dots in the retinal mid-peripheral region (79%); MULIBREY nanism in a sibling in 17%. Minor signs include peculiar high-pitched voice (96%), hepatomegaly (70%), cutaneous naevi flammei (65%), and fibrous dysplasia of long bone (25%) [65].

Cardiovascular manifestations include both congenital and progressive ones. Right-sided HF (50%) is often observed along with signs of pericardial calcium deposition on radiographs. ECG changes include prominent P waves and flattening/inversion of T waves. Abnormal ventricular and supraventricular arrhythmias, including Wolff–Parkinson–White (WPW), have also been frequently recorded. Pericardial calcification, a hallmark of this disease, leads to constrictive pericarditis. The fibrotic/calcific process extends into the myocardium and can lead to end-stage heart failure (ESHF). Treatment options include pericardiectomy and simultaneous removal of any calcific lesions. HT is disputed due to multisystemic comorbidity, which is variable in nature and includes different organs [66,67,68].

### 2.3. CMP + Limb Defects 

The specific disorders reported in this paragraph is summarized in Figure 4.

#### 2.3.1. Holt–Oram Syndrome (HOS)

Holt–Oram syndrome (HOS, MIM # 142900) is a rare AD condition also known as “Heart-hand syndrome”. In about 70% of cases, HOS is caused by pathogenic variants of *TBX5* (OMIM # 601620) coding for T-box Transcription Factor 5 and located on 12q24.21 region [69]. TBX5 is involved in the regulation of cell types, mainly cardiomyocyte differentiation, morphogenesis, and organogenesis [70].

HOS is characterized by high intra- and inter-familial variability of upper-limb defects (ULDs) (in up to 90%) and cardiac abnormalities (75%) in terms of CHDs, arrhythmias and CMP. Recently our group has reviewed the literature data corresponding to the wide cardiac phenotypic spectrum related to *TBX5* pathogenic variants [18].

The ULDs can be very variable: unilateral or bilateral, symmetric or asymmetric (mainly radial defects). The most common malformations include radial ray defects such as triphalangeal or absent thumb (s), phocomelia, fusion or anomalous development of the carpal and thenar bones, abnormal pronation and supination, abnormal opposition of the thumb, sloping shoulders and restriction of shoulder joint movements [18].

The major three components of *TBX5* cardiac expression embrace: a. CHDs both typical (ASD, VSD and PDA) and atypical (complex CHDs such as HLHS, total anomalous pulmonary venous return (TAPVR) and TA and other conotruncal malformations as TOF and AVCD [71,72]; b. arrhythmic spectrum, which was traditionally described as a progressive atrioventricular block (AVB). However, recent evidence brings light to tachyarrhythmias (both supra ventricular and ventricular ones), including Long QT syndrome (LQTS) and Brugada Syndrome (BrS) [73,74]; c. the third component includes limited cohorts of patients with CMPs, mainly DCM. Animal model studies report the predisposition of *TBX5* knock out mice to DCM. It is important to note that this myocardial dysfunctional susceptibility appears independent from CHD related hemodynamic causes predisposing to HF [75,76].

#### 2.3.2. CACNA1C-Related Disorders

*CACNA1C* (OMIM # 114205) encoding CaV1.2 is a calcium channel located on the 12p13 region with an important role in the development of the action potential in human cardiomyocytes. It is involved in a complex pattern of phenotypic expression. To date, around 100 patients are reported with pathogenic *CACNA1C* variants, which incorporate both isolated cardiac and less frequently multisystemic disease. In fact, the clinical spectrum related to *CACNA1C* variants is highly variable [77].

The cardiac spectrum includes three major components: arrhythmic (major), CHDs (TOF, PDA and VSD), and myocardial changes (HCM and rarely LVNC) [77,78].

*CACNA1C* related arrhythmias encompass majorly LQTS type 8, less frequently BrS and ventricular arrhythmias. In a recent study, the role of *CACNA1C* variants in short QT syndrome (SQTS) has been disputed and might not be considered anymore in this specific subtype [79].

In original reports, *CACNA1C* variants were related to the multisystemic condition known as Timothy syndrome (TS, MIM # 601005), which is characterized by a variable association of cardiac involvement (as abovementioned), cutaneous syndactyly (fingers and toes), craniofacial anomalies (depressed nasal bridge, low-set ears, thin vermilion of the upper lip, round face, and abnormal tooth development), neurological and/or/neuropsychiatric manifestations (seizures, hypotonia, behavioral anomalies), and recurrent infections [80].

The most frequently reported pathogenic *CACNA1C* variants are in exon 8 p.Gly406Arg and p.Gly402Ser [77]. Literature revision shows high phenotypic variability related to these two variants ranging from isolated cardiac to multisystemic involvement. Up to date, there are no genotype and phenotype correlation studies and future multicentric studies are needed to delineate this condition appropriately.

### 2.4. CMP + Progressive Neuromuscular Disease + Delayed Motor Milestones 

The specific disorders reported in this paragraph is summarized in Figure 5.

#### 2.4.1. Friedreich’s Ataxia (FRDA)

Friedrich’s ataxia (FRDA, MIM # 229300-601992) is a monogenic disorder spinocerebellar ataxia with a prevalence of 1–9/100,000. FRDA is an AR disease with a pathogenetic mechanism related to biallelic abnormal triplet repeat expansion of the GAA in the first intron of the gene encoding frataxin (*FXN*) located on chromosome 9q21.11 [81].

FRDA’s main clinical manifestations (75%) include progressive ataxia of the limbs and trunk, lack of deep tendon reflexes, sensory disturbances, skeletal deformities, diabetes, and cardiac involvement [82]. About 25% show atypical presentations (late-onset or retained tendon reflexes) [83].

Patients can show initially poor balance when walking. Children might be defined by parents as clumsy toddlers, slow walkers, those who have difficulties in going up and downstairs, inability to ride a bike. In the early years of life, no clear neurological defect can be noticed [84]. Other signs include slurred speech, upper-limb ataxia, gait ataxia and poor balance especially accentuated when visual input is eliminated (Romberg sign) [83].

Autonomic disturbances become more common with disease progression, including cold, cyanosed feet [83].

CMP in FRDA is observed in 57–81% of cases [85]. Some recent reports show prevalent concentric HCM with both subclinical diastolic and systolic dysfunction in children [86]. FRDA represents 8.6% of childhood-onset HCM [48,49,50,51,52,53,54,55,56,57,58,59,60,61,62,63,64,65,66,67,68,69,70,71,72,73,74,75,76,77,78,79,80,81,82,83,84,85,86,87].

Child et al., in 1986, suggested two possible aspects which lead to HF in FRDA: a “dystrophic” type with a prevalence of tissue fibrosis and a “hypertrophic” type, as concentric or asymmetric myocardial hypertrophy [88].

Rarely, asymmetric septal hypertrophy, an LV outflow gradient, and ESHF with DCM secondary to HCM may occur [82,84].

In a pediatric population, ECG disturbances may be secondary to myocardial fibrotic changes [89,90].

Recently our group reviewed cardiac manifestations of selected neuromuscular diseases, including FRDA. We summarized major clinical, management and therapeutic options in these rare conditions [91].

#### 2.4.2. Kearns–Sayre Syndrome (KSS)

The Kearns–Sayre syndrome (KSS, MIM # 530000) is a rare (prevalence 1–9/100,000) heterogeneous neurodegenerative disorder. In the majority of cases, it is caused by a single large heteroplasmic deletion of the mitochondrial DNA (mtDNA) of 4.977 bp, known as m.8470_13446del4977. Rarely other pathogenic variants in either mtDNA or nuclear genes are related to KSS [92].

The clinical features of KSS encompass musculoskeletal, central and peripheral nervous, endocrine and cardiac involvement. The main characteristics are progressive external ophthalmoplegia and retinitis pigmentosa with onset before the age of 20 years with at least one other sign: cerebellar ataxia, cerebrospinal fluid (CSF) proteins > 100 mg/dL and cardiac conduction disturbances [93].

Cardiac involvement includes structural (valvular and myocardial) and arrhythmic (both tachy- and brady-arrhythmic) anomalies.

The main neuromuscular aspect of this disease involves extraocular muscles (ophthalmoplegia and ptosis) and facial muscles in some patients. Involvement of the orbicularis oculi muscles can cause difficulty in tightly closing the eyelids, or weakness of the frontal muscle leads to difficulty in opening the eyelids. Impairment of chewing muscles can lead to dysphagia. In the late stages, progressive weakness of the neck, shoulder and extremities muscles can be observed [94].

Cerebellar ataxia, sensorineural hearing loss, neuropathy, and impaired intellectual function are other possible signs [94].

Rare multi-valvular involvement, as mitral and tricuspid valve prolapse, is observed in children [95,96,97], and a single report of a teenager with KSS diagnosed and dilated aortic root without other risk factors contributing to these data [98].

The arrhythmic aspect in KSS is a major prognostic aspect since progressive cardiac conduction defects up to complete AVB, LQT, supra-ventricular tachyarrhythmias and ventricular tachyarrhythmias are described with syncope in about 50% of patients. Sudden cardiac death (SCD) is reported in 20% [99,100,101].

CMP is described in studies but frequently without a specific myocardial pattern [91,95,102,103]. HF is reported in the late stages of KSS but rarely in children [97,104,105,106,107].

#### 2.4.3. Danon Disease (DD)

Danon disease (DD, MIM # 300257), also termed glycogen storage disease IIb, is a rare X-linked condition caused by deleterious variants in *LAMP2* (lysosome-associated membrane protein 2, OMIM # 309060). LAMP2 has an important role in lysosomal fusion. Its dysfunction leads indirectly to the inability to remove aged mitochondria (mitophagy) with subsequent mitochondrial dysfunction, energetic deficiency, and abnormal oxidative stress. DD can be an example of direct and indirect autophagy disorder. DD is apparently rare, but its geographic distribution is wide [108,109].

It is a multisystem disease with predominant involvement of the heart, skeletal muscles, retina and cognitive ability. Due to the X-linked nature of the disease, males are typically more severely affected and show multiorgan and earlier manifestations than females [110].

Extracardiac findings consist in a variable degree of skeletal muscle weakness 60% in males versus 3% in females (which can lead to delayed motor milestones); variable degree of ID (73% of males and 9% of females), and variable degree of retinopathy (20%) with visual impairment. Cone dystrophy in DD can be of variable severity that might be underestimated [111,112,113,114]. In females, retinal findings appear to be milder than those seen in males [112,114,115,116]. The outcome in females is unclear, but changes in the peripheral retinal pigment epithelium can lead to the near loss of visible pigment, abnormal electroretinogram, and impaired vision [111].

Blood tests can represent a red flag leading to the diagnosis of this condition due to the frequent finding of increased creatine phosphokinase (CPK) in 80% of males but not in women and elevated liver transaminase enzymes in 83% of males but in 6% of females.

Cardiac manifestations (in up to 96% of patients) seem to be the major contributing factor of major comorbidity and mortality in this condition. Early penetrance is common, and a poor prognosis is often expected [117].

Abnormalities in cardiac conduction are very frequent. It can be associated with tachyarrhythmia (both supraventricular and ventricular) and bradyarrhythmia. These abnormalities range in WPW patterns, which can proceed HCM (41% females vs. 50% males); first-degree AVB, advanced AVB (15%), paroxysmal or permanent atrial fibrillation (common in females 55% versus 27% in men), malignant ventricular tachyarrhythmia (sustained ventricular tachycardia, ventricular fibrillation, or SCD) occur in both genders. An implantable device is described in 67% of patients [110,111,112,113,114,115,116,117,118].

CMP is present in 96–100% of affected males. LV hypertrophy is observed in around 75% of both males and females. HCM can be obstructive in 18% of males but very rarely in females. Hypokinetic CMP or end-stage DCM was found at the time of presentation in 40% and 59% males and females, respectively, with a mean ejection fraction of 34 ± 11% and 28 ± 13%, respectively [110]. Myocardial dysfunction is often severe, progressive, and often requires HT [111]. The outcome appears the same in both genders, but complications manifest at altered ages in females [112]. An important issue in the peri-transplant phase includes difficulty in recovery for respiratory, motor and potential predisposition to infections (personal observation). These complications might be related to underlying constitutional muscular weaknesses that become influenced by prolonged immobility, immunosuppressive treatment, and mitochondrial dysfunction. These data need to be confirmed in larger studies.

### 2.5. CMP + Skin+ Hair 

The specific disorders reported in this paragraph is summarized in Figure 6.

#### 2.5.1. Naxos Disease

Naxos Disease (MIM # 601214) is an ultra-rare monogenic AR disease characterized by childhood-onset diffuse palmoplantar keratoderma (PPK), wooly hair and right ventricular (RV) ACM. It is caused by biallelic variants in the plakoglobin gene (*JUP*, OMIM # 173325) [119,120]. In Naxos Disease, more than 90% of the reported individuals carry *JUP* 2157del2 variant [121].

Major hair changes include peculiar, wooly, dense and sparse abnormalities [122,123,124]. Partial alopecia is also observed variably involving eyebrows [125,126].

On palms and soles, keratotic lesions can be observed in variable severity, progressivity, and distribution within the affected family members and for the same individual at different seasons of the year [127]. Skin is eczematous and fragile with possible erosions and ulcers on the perioral and sacral region or dorsal surfaces of the hands and legs. These changes are frequent in early infancy, with improvement in later childhood. Diffuse hyperkeratotic palmoplantar plaques that develop later have a well-demarcated erythematous border [128]. Infrequently, less pronounced hyperkeratosis can be seen over the flexor and extensor surfaces of the joints and the perianal and intergluteal folds [125].

Regarding cardiac involvement, one-third of patients become symptomatic before the age of 30 years [129]. One of the earliest signs can be frequent PVC of RVorigin and depolarization changes in asymptomatic children with normal myocardial imaging findings [130].

ACM is often associated with major arrhythmic events, including sustained ventricular tachycardia, left-bundle branch block leading to syncope and occasionally SCD [122,129].

Other resting ECG changes include T-wave inversion in V1–V3 or across the precordial leads and Epsilon waves in almost 38% of patients [127].

ACM in Naxos shows 97% penetrance in adolescents [131,132]. Echocardiographic findings may vary, ranging from mild dilatation of the RV and regional hypokinesia to severe dilatation and diffuse dysfunction. LV dilatation and hypokinesia have been additionally reported in some patients [133,134]. Structural or functional changes of RV are documented in all adult patients [127]. LV involvement has also been detected in one-third of patients at presentation [127].

Myocardial tissue changes of affected individuals show myocyte loss and fibrofatty replacement of the RV myocardium and inflammatory infiltrates, which may mimic and be misdiagnosed as acute myocarditis [135]. Major RV myocardial involvement is noticed in the subepicardial and mediomural layers, regionally being transmural, with aneurysmal formations. Surviving myocytes are surrounded by fibrous tissue embedded within fatty tissue. The LV is variably involved, from regional fibrotic replacement [130] to extensive transmural myocardial atrophy and fibrofatty replacement [136].

#### 2.5.2. Carvajal Syndrome (CS)

Carvajal syndrome (CS, MIM # 605676) is another ultra-rare monogenic disease caused by pathogenic variants in the desmoplakin gene (*DSP*, OMIM # 125647). The condition is AR for the full-blown picture of the ectodermal-cardiac disease with biallelic pathogenic *DSP* variants. Monoallelic/single heterozygous pathogenic *DSP* variants can be responsible for ACM with (MIM # 615821) or without (MIM # 607450) hypo/oligodontia but also associated with PPK and wooly hair [121,133,137,138,139,140,141], Norgett et al., 2000.

Some authors consider CS as a variant of Naxos syndrome [142].

In fact, ectodermal phenotype includes a variable degree of wooly hair, striate PPK, additional dental abnormalities and leukonychia [143,144,145]. Wooly hair is present from birth, while PPK develops after infancy [143].

Common ECG changes in CS are low voltage and intraventricular conduction defects and T-wave V1, V2 or V3 or V5 inversion [146,147].

Cardiac structural involvement shows early onset asymptomatic LV dilatation. It might be overlooked without purposely scheduled cardiac imaging. In adolescents, ACM is generally rapidly progressive, leading to extreme LV dilatation (90%), HF or SCD [143,148]

#### 2.5.3. Menkes Disease (MD)

Menkes Disease (MD, MIM # 309400) is an X-linked recessive disorder of copper metabolism caused by pathogenic variants in *ATP7A* (OMIM # 300011) with an incidence of 1 in 300,000 in Europe [149,150], but it results less frequently in Japan and more frequent in Australia probably due to a founder effect [151,152].

MD has a severe clinical course. Mortality generally occurs in early childhood. Occipital horn syndrome (OHS) is a mild form of the disease, but there are also other intermediate forms [153].

The main findings of MD include progressive neurodegeneration, connective tissue disturbances and peculiar ‘kinky’ hair [153]. Females are usually asymptomatic or with an attenuated form of the disease [154,155].

Hair abnormalities include a hypopigmented/depigmented aspect, steel wool-like appearance, lusterless and friable nature, especially in the areas of the scalp subjected to friction [153]. At light microscopy, hair shafts show twisted aspects around their own axes (*pili torti*), with varying shaft diameters (*monilethrix*), and fragmentation at regular intervals (*trichorrhexis nodosa*) [156].

Other possible signs in these patients are cerebral/cerebellar neurodegenerative and connective tissue anomalies. At the age of three months old, patients begin to lose skills, with regression of motor functions, seizures, hypotonia, skin laxity, jowly appearance with sagging cheeks, frontal bossing, and vascular tortuosity [157,158].

CHDs are described sporadically in this condition, including TOF, transposition of the great arteries (TGA), pulmonary stenosis, CoAo, and complete congenital AVB. Literature revision shows that cardiac involvement can reach up to 4.2%, but it might be underestimated [159,160].

CMP is not directly reported as associated with MD in humans, but experimental animal studies reported a possible correlation of copper metabolism with CMP [161]. In a personal observation, we have diagnosed a family with two affected males with early-onset CMP with the full-blown picture of the disease and a carrier mother (personal observation—Baban, 2021).

#### 2.5.4. Cantù Syndrome (CS)

Cantù syndrome (CS, MIM # 239850) is a rare AD disorder caused by heterozygous gain-of-function variants in *ABCC9* (97%, OMIM # 601439) and, rarely, in *KCNJ8* (1–2%, OMIM # 600935), encoding the regulatory (SUR2) and pore-forming (Kir6.1) subunits, respectively, of ATP-sensitive potassium (KATP) channels [162].

It is characterized by a variable constellation of signs and symptoms that can reach almost any organ with variable expressivity, including hypertrichosis (thick scalp and excessive body hair) which is observed since birth in 99% of patients. Other cutaneous manifestations include wrinkled and/or loose skin (50%), visible veins (50%), and joint hypermobility (56%). It is characterized by a coarse facial appearance with a low frontal hairline, epicanthal folds, flat nasal bridge, wide mouth, full lips, macroglossia, persistent macrocephaly, and generalized macrosomia. Skeletal abnormalities, including osteochondrodysplasia and generalized osteopenia, are observed later in life. Developmental delay is variable, but intellect is typically normal. However, behavioral problems are considered an emerging issue. Other features include gastroesophageal reflux (42%), intestinal dysfunction (17%), renal problems (14%), immune dysfunction (41%) with recurrent respiratory infections, frequent myopia and/or hyperopia, hearing loss (33%) and other minor entities [163,164].

Based on the latest reports and on the largest series that describe CS in a multidisciplinary manner, there is the fact that shows that the cardiovascular manifestations in CS are one of the most variable ones with the widest spectrum that can stand from a static malformative aspect to progressive dysfunctional or vascular ones. It can encompass: a. CHDs 75% (PDA 58%, ASD, PFO, VSD), b. valvular defects 25–30% (mitral valve regurgitation, aortic stenosis, BAV), c. dilated aortic root and ascending aorta with a rare aortic aneurysm 32%, tortuous vascularity involving brain and retinal vasculature where the true percentage is unknown in the entire population; d. pulmonary hypertension in 24%; e. pericardial effusion in 25–30%; and f. cardiomegaly (HCM and DCM) 61%; g. arrhythmias are described in 24% of cases but are of unspecified nature.

Edema was observed in almost half of the patients, which is an independent observation from HF status in this syndrome. Edema in CS is self-limiting in some patients or needs specific therapeutic measures, including compression or diuretics. HF is observed in 10–15% of patients, but neither outcome, indication to assist device, nor HT were ever mentioned in the literature [163,164].

#### 2.5.5. Epidermolysis Bullosa (EB) Simplex Secondary to KLHL24 Variants

Inherited epidermolysis bullosa (EB) is a rare heterogeneous disorder characterized by cutaneous and mucosal fragility. In 2016, a specific subgroup of EB simplex was described, which is caused by the gain of function variants in *KLHL24* (OMIM # 611295), encoding a protein involved in ubiquitination [165,166,167]. Both AD and AR forms are reported.

Major cutaneous manifestations include skin defects and blistering at birth and unusual stellate scarring, skin fragility, and whorled or macular hyperpigmentation or hypopigmentation in childhood. Skin fragility improves by adulthood, but nail dystrophy, anetoderma, and hair loss may persist.

Recent reports in studies show progressive cardiac involvement from the second decade of life, including arrhythmic events (majorly tachyarrhythmias and less frequently bradyarrhythmias). Myocardial involvement varies from DCM (in AD form) and HCM (in AR form). Other emerging cardiac findings include increased cardiac biomarkers (encompassing brain natriuretic peptide –BNP-, troponin, and CPK) and extensive late gadolinium enhancement (LGE) changes on CMR with transmural fibrosis, leading to thinned ventricular walls [165]. Cardiac involvement is thought to reach 40% of patients from the second decade, but future studies are needed to derive a definitive conclusion. A major limitation is due to the relatively new description of the disease and indication to genotype-phenotype correlation studies in order to determine risk stratification and management plan of this rare condition.

### 2.6. CMP + Eye 

The specific disorders reported in this paragraph is summarized in Figure 7.

#### Alström Syndrome (AS)

Alström syndrome (AS) (MIM # 203800) is a monogenic AR disorder with a prevalence of 1–10 per million, and it is caused by biallelic variants in *ALMS1* (OMIM # 606844) [168,169]. AS is characterized by progressive and early multiorgan involvement that encompasses retinal dystrophy and blindness, hearing loss, obesity (80–100%), diabetes mellitus type 2 (DM2) (20–69%), hypertriglyceridemia (40–77%), hypercholesterolemia (15–36%), essential hypertension (40%), and CMP. Diffuse interstitial fibrosis is the commonest multiorgan sign in AS; it can involve kidneys, pancreas, liver, lungs, and the heart, as well as other organs. Life expectancy is rarely over 50 years of age [168,169].

A typical sign of AS is early visual dysfunction, nystagmus, photophobia or light sensitivity, often with complete blindness in the second decade [170,171]. However, there is some variability in the age of onset and rate of progression. Bilateral subcapsular cataracts and retinal changes in infancy such as attenuation of retinal vessels, pallor and atrophy of the retinal pigment epithelium are also common [171,172,173,174].

Sensorineural hearing loss is variable in the age of onset and severity. However, it is often described in up to 89% of patients, with an onset within the first decade of life, progressive in nature, and it can reach moderate to severe degrees [170,175,176]. In some cases, chronic and acute otitis media represent a contributing factor to the degree of hearing loss with a component of the conductive part [169].

Regarding cardiovascular manifestations in AS, ECG abnormalities are variable, encompassing left axis deviation, non-specific T wave changes, and poor R wave progression. QT prolongation is described [168,169,170,171,172,173,174,175,176,177]. However, patients do not seem to show an increased incidence of major arrhythmic events [168].

CMP is a typical finding of AS. DCM and HF manifest in approximately two-thirds of patients [170]. Another subset of individuals developed adult or adolescent-onset HF caused by myocardial hypertrophy and dilation with restrictive impairment in both ventricles [178,179].

In the cohort reported by Marshall et al., three subgroups were described: 39% did not show CMP (age range 2–33 years); 43% had infantile-onset CMP, and 18% showed later-onset CMP. In infantile CMP, Marshall et al. mentioned apparent recovery of cardiac function within the first years of life. However, these data were not confirmed by a large study driven by Brofferio’s group [168,169].

Subclinical CMP might be triggered by secondary factors and manifest with overt HF [168,169,170,171,172,173,174,175,176,177,178,179,180]. It is important to remember the several comorbidities in AS that can predispose to myocardial changes, including obesity, DM2, and dyslipidemia. Even in patients with an apparent absence of CMP, strains can be abnormal, which can represent a substrate to underlying myocardial changes.

CMR studies are limited. However, they report a reasonable percentage of peculiar myocardial changes, including patchy and or diffuse intramyocardial fibrosis in LGE studies differently from ischemic LGE changes [168,169,178,181]. The true mechanism and timing of intra-myocardial fibrosis are still unknown. Multifactorial origin is probably not only due to the genetic background of biallelic *ALMS1* variants but also due to obesity, DM2, activation of the renin-angiotensin-aldosterone system and dyslipidemia [182,183].

All the reported factors can have an important role in late-onset CMP in AS. However, it is difficult to explain them in relation to the acute infantile CMP that appears to have a separated entity [168].

In this severe and rare disease, multicentric studies are needed to derive conclusions and delineate an appropriate cardiac management strategy.

### 2.7. CMP + Corpus Callosum Agenesis 

The specific disorders reported in this paragraph is summarized in Figure 8.

#### Vici Syndrome (VS)

Vici syndrome (VS, MIM # 242840) is a very rare disease (so far less than 100 reported patients) due to AR pathogenic variants in *EPG5* (OMIM # 615068) with a key role as a regulator of autophagy in multicellular organisms [184,185].

Its incidence is unknown, an exponentially increasing number of patients are reported in studies, but it seems largely misdiagnosed [186].

VS is a severe congenital multisystem disorder, with an onset in the first months of life. It is characterized by the principal diagnostic features of agenesis of the corpus callosum 97.4%, Profound developmental delay 97.4%, failure to thrive 30.8%, oculocutaneous hypopigmentation 96.2%, cataracts 62.8%, progressive microcephaly 57.7%, CMP 65.4%, combined immunodeficiency 76.9% with recurrent infections 98.7% and other variable signs such as hypotonia 47.4%, and epilepsy 33.3% [186]. Data in studies show that virtually any system can be involved, including hearing loss, thymus aplasia or hypoplasia in 20%, hepatomegaly with or without dysfunction 10%, renal abnormalities both structural and functional 15% [185,186,187].

Agenesis of the corpus callosum is one of the five principal diagnostic features [186], but other CNS findings consist in pontine hypoplasia, reduced opercularization of the Sylvian fissures, delayed myelination and generalized reduction in white matter [186,187]. Other less commonly observed CNS abnormalities are cortical malformations, cerebellar abnormalities and abnormal signaling within the thalami, similar to lysosomal storage disorders [188].

Cardiac involvement in VC is a major sign that is observed in up to 90% of patients [186]. Literature revision shows an absence of a study that systematically takes this aspect as a major focus.

CHDs (mainly ASD) are reported in around 10% of patients. PFO is described in VS. However, it is a frequent finding within the general population [186].

CMP is a major sign that reaches 80% of patients [186], with a variable age of onset (infantile to late childhood). Intercurrent illnesses such as infections can be triggering factors for acute HF [185].

Since the original report, CMP is mentioned as a major criterion in VS. However detailed and specified subtype description is not available in studies. In other words, no study reports the natural history of cardiac involvement nor potential arrhythmic changes in these patients. Both HCM and DCM are reported [186], but it is unclear whether the DCM is a spectrum of an end-stage HCM similar to other autophagy disorder related conditions similar to Danon disease.

Few detailed histological illustrations are available in studies. In two unrelated patients where postmortem examination was performed [189,190], major LV structural changes included variable degrees of interstitial fibrosis, abnormal cardiomyocytes containing vacuoles and membrane-bound cytoplasmic inclusions, possibly with glycogen [186,187,188,189].

### 2.8. CMP + Recurrent Infections 

The specific disorders reported in this paragraph is summarized in Figure 9.

#### Barth Syndrome (BTHS)

Barth syndrome (BTHS, MIM # 302060) is an X-linked recessive disease caused by deleterious variants in *TAZ* (OMIM # 300394) [191]. In BTHS, less mature cardiolipin is produced, which is an important component for high energy-requiring tissues such as cardiac muscle [192]. Cardiolipin is involved in maintaining mitochondrial structure and super complexes. Mitochondrial integrity is fundamental for different cellular processes enclosing hypoxia, apoptosis, autophagy, cell proliferation, and differentiation [193].

BTHS frequency is about 1/300,000 to 400,000 births, and there are also reports on a substantial risk of fetal loss and stillbirths [194,195]. There is no known racial or ethnic predilection and emerging evidence that it is highly underestimated [196]. Nowadays, there are no genotype/phenotype correlation studies. BTHS shows high intra- and inter familiar variability [197]. Differently from Danon syndrome, this X-linked is less represented in female carriers.

BTHS is principally characterized by skeletal myopathy (97%), CMP (73%), neutropenia (70–85%), growth retardation/delay in puberty (58%) [198], and increased levels of 3-methylglutaconic aciduria (3-MGC). The latter biomarker is reported in 88% of patients [199], and the defective phospholipid remodeling is expressed by elevated levels of the monolysocardiolipin/cardiolipin (MLCL/CL) ratio in blood spot and abnormal cardiolipin profiles in lymphocytes and tissues [200,201]. 3-MGC levels can be normal on single sample testing [202] and may be normal on repeated urinary tests for the first 6 to 18 months of life [203]. In the latter study performed by our group, we recommend proceeding to molecular testing and/or repeating, periodically, this specific biomarker testing when clinical suspicion is high due to “incomplete penetrance” of 3-MGC release in all affected patients.

The mortality related to BTHS is high throughout infancy, which is primarily related to progressive CMP and immune deficiency [191].

Neutropenia is a highly variable feature in BTHS. It can be the presenting feature, manifest late; it can be absent, mild, persistent, intermittent, truly cyclic, and unpredictable [196,197,198,199,200,201,202,203,204].

Regarding the cardiac aspect, it has two major manifestations: the arrhythmic spectrum and myocardial changes.

It can be associated with ventricular arrhythmias and SCD independently from structural myocardial anomalies [205,206,207]. Ventricular arrhythmia is documented in 13% of patients necessitating an implantable cardioverter-defibrillator (ICD) [208] in older children [196]. Prolonged or borderline prolonged QTc is observed in 43% of patients [198] with or without myocardial involvement [209].

A major clinical feature in BTHS is CMP, with high prevalence in early life. In 70% of patients, CMP occurs in the first year of life [208]. In many affected individuals, CMP improves after initial infantile-onset or stabilizes with anti-HF medical treatment in toddlers [210] and personal observation.

The myocardial phenotype shows DCM in the majority associated with endomyocardial fibroelastosis [205]. LVNC is described in 50%. Instead, HCM is rarely observed [198,199,200,201,202,203,204,205,206,207,208,209,210,211]. In the same case, both DCM and HCM are seen in the same family [198], and overlapping myocardial features can be detected in the same individual [212,213]. HT is successfully reached in BTHS, especially when immunological and systemic comorbidities are less expressed [203].

### 2.9. CMP + Tall Stature 

The specific disorders reported in this paragraph is summarized in Figure 10.

#### Marfan Syndrome (MFS)

Marfan syndrome (MFS, MIM # 154700) is an AD condition characterized by multisystem involvement. The main signs are generally cardiac, ocular, and skeletal, but any other organ or system with connective tissue background can be affected. In most individuals, a pathogenic variant is identified at *FBN1* (OMIM # 134797), located on 15q21 and encoding with Fibrilin-1 [214]. In 90% of MFS patients, an *FBN1* variant is detectable through sequencing analysis. On the other hand, 5% of cases are due to deletions or duplications in one to more exons of the gene, detectable through multiplex ligation-dependent probe amplification (MLPA) [214].

Diagnostic criteria follow the 2010 revised Ghent Nosology, taking into consideration the major organ involvement in this condition [215]. However, it is important to take into consideration that the disease penetrance is age-related. In other words, final judgement of suspected cases in children must not be conclusive due to the dynamic nature of the disorder and the possibility to express certain criteria later in life (often before 20 years old).

From a multisystemic point of view, a major sign is represented by *ectopia lentis* (EL), reaching up to 50% of patients. Other signs include different degrees of myopia, including severe forms. A variable combination of musculoskeletal features can be observed, including tall stature (not pathognomonic in MFS), dolichocephaly, pectus excavatum or carinatum, scoliosis, arachnodactyly and other features, also known as Marfanoid habitus [215].

Revised Ghent criteria include a combination of different multisystemic features and family history for MFS.

The major cardiac sign related to MFS is represented by aortic root dilatation leading to aneurysmatic progressive changes and susceptibility to subsequent rupture. Aortic root dilatation is estimated by echocardiographic measurement of aortic diameter (Ao) at the sinuses of Valsalva above indicated Z-score or aortic root dissection. The 2010 revised Ghent Nosology takes into account aortic changes for the establishment of MFS diagnosis on the basis of two main groups. In the first group, when family history is negative, MFS is confirmed if Ao (≥2) is associated with EL or FBN1 variant or systemic score (≥7 points). In the latter group, other conditions in differential diagnosis to MFS must be taken into consideration. The second group is in the presence of positive family history for MFS when AO (Z score ≥ 2 above 20 years old, ≥3 below 20 years). In the absence of identified FBN1, other conditions in differential diagnosis to MFS must be taken into consideration [215].

Valvular involvement in MFS is well represented. Mitral and tricuspid valves may be implicated with prolapse and regurgitation. Excessive proximal pulmonary artery dilatation is observed in a reasonable percentage of patients.

A few years ago, we revised in a comprehensive manner the medical management of MFS and related conditions, bringing light on the latest medical and surgical options related to MFS [216].

Recently Mosquera and De Backer (2021) have described possible myocardial changes in the form of CMP in MFS. They report two different clinical entities: HF in neonatal MFS (nMFS) and CMP in classical MFS. Children with nMFS generally show tricuspid and mitral valve prolapse, usually with severe progressive regurgitation leading to congestive HF [217].

Moreover, several (historical) series on survival in MFS have listed HF as one of the leading causes of death and indication for HT. HF in MFS can reach up to 30% [218,219], which might lead to the risk of aortic dissection. Initially, myocardial dysfunction is mostly mild and subclinical and does not progress rapidly. One of the major triggering factors for HF in MFS is the underlying severe valvular disease (aortic and/or mitral valve regurgitation). However, several reports from independent researchers have shown intrinsic myocardial dysfunction in MFS. In fact, the reported prevalence of what is now known as Marfan CMP ranges from 3% [220] to 68% [221] across different series, depending on the definition and population characteristics. The involvement of both left and right ventricles with systolic and diastolic dysfunctions are well described [222,223]. A possible link between intrinsic CMP and an unfavorable course in the event of an additional hemodynamic trigger such as valvular dysfunction and/or aortic root replacement has not yet been demonstrated but seems plausible.

Major and progressive hemodynamic changes may trigger myocardial fibrosis [224]. In a single study, children with MFS and Loeys–Dietz syndrome (LDS), another related connective tissue disease, showed increased LV and RV volumes and diffuse myocardial fibrosis on CMR compared to healthy individuals [225]. Further studies are needed to establish more solid results. Genotype–phenotype correlation studies are not available except for two independent observations where a higher incidence of LV dilatation and decreased LV function in patients carrying non-missense variants [226,227,228].

### 2.10. CMP + High Inbreeding

In clinical practice and in large pediatric cohorts, clinicians face complex clinical pictures of children coming from a background with a high inbreeding rate. This fact leads to a high prevalence of AR disorders in consanguineous populations. Examples of inbred communities are Middle Eastern, Asian (such as from Pakistan), and Gypsy families. Other highly inbred populations include those coming from geographical isolation (Finland, Iceland and Sardinia Islet in Italy).

Despite the fact that consanguineous marriages are discouraged by the major religions, recent studies estimated its prevalence among the Middle Eastern population is up to 40%, representing some of the highest rates in the world [229,230,231]. This high inbreeding rate leads to a high prevalence of private and ultra-rare AR disorders.

Certain populations can have a higher frequency of otherwise rare conditions. One of these examples is *TMEM70* (OMIM # 612418) related disorders in the highly inbred ROMA ancestry due to the c.317-2A>G founder mutation [232]. *TMEM70* is an AR mitochondrial disease (MIM # 604273) characterized by oligohydramnios (40%), IUGR (47%), prematurity, CMP (89%), microcephaly (71%), facial dysmorphisms (66%), lactic acidosis, hyperammonemia, hyperuricemia and increased secretion of 3-MGC (95%) [199]. The latter biomarker can be missing or intermittent in some patients [203].

Cardiac involvement in *TMEM70* related disorder includes variable degree and age of onset of persistent pulmonary hypertension (22%), WPW pattern (13%), and CMP (more frequently HCM and less frequently DCM).

In this review and according to progressively growing evidence in studies and in our personal experience (unpublished data), we recommend a first-line exome study in children born to consanguineous marriage. The recommended strategy is homozygosity mapping combined with exome sequencing for genetic analysis in order to avoid time-wasting with custom panels due to higher rates of private conditions that can be easily overlooked with conventional limited next-generation sequencing (NGS) studies.

In the field of complex systemic CMP in children, clinicians can face unconventional manifestations of certain ultra-rare diseases. In other words, CMP can be the expanding phenotype/sign and the previously unreported finding in certain ultra-rare conditions. This emerging issue can be explained due to the rapidly increasing global description of previously limited cohorts and due to the fact of prolonged survival of these children leading to the appearance of unpredictable signs. In order to explain this vision, we report the example of a toddler who is the first child of a highly inbred Pakistani family with the manifestation of repeated respiratory distress and infections that needed tracheostomy with subsequent stenosis. She showed developmental delay, neurosensory bilateral hearing loss, strabismus, diffuse large bullous skin lesions partially leading to erosion and eruption of the skin majorly in skin folds (neck, axilla, elbows, genital, and knees). At the age of 2 years and a half, echocardiography showed LVNC with normal LV function and dimensions. She had a rapid worsening of clinical condition after a triggering factor of urinary tract infection. Subsequently, she developed cerebral edema and aborted SCD. After a few days, cerebral death was declared due to irreversible brain damage. Clinical exome study showed homozygous variant in *NAXE*: c.608_616delTCCTGAGTG (p.Leu204_Val206del) and confirmed the diagnosis of AR *NAXE* gene mutation-related encephalopathy, a lethal neurometabolic disorder. In this specific case, we believe that the myocardial manifestation of LVNC can be an emerging sign or phenotypic expansion of this lethal disease.

## 3. Complex Multisystemic CMPs and Autophagy

During the process of literature revision, we have perceived a “repeating pattern” of a relatively common etiologic component in some forms of complex/multisystem CMP. In other words, certain syndromes such as Danon, Vici, Alström, Barth, and Myhre syndromes’ share a common pathway of directly or indirectly defective “autophagy” process. Autophagy represents a fundamental multi-step mechanism that maintains homeostasis through the removal of defective proteins and organelles, defence against infections and adaptation to changing metabolic demands [233,234]. This process is particularly enhanced in neurons, muscles and probably in the heart. In most of these disorders, the major final step appears to be defective autophagy and fibrosis with possible initiating/triggering factors for CMPs. This conjoint aspect in all these rare diseases could be important for possible prognostic/therapeutic implications in this category of patients. However, multicentric studies detailed functional and experimental models are needed prior to deriving conclusions.

## 4. A Link between CHDs and Children CMP

A final aspect to focus on is the association of CMP with CHDs and arrhythmias that were previously thought to be separated entities. This watertight is slowly vanishing, and previously determined barriers separating these three major groups seem to be inconsistent. Several complex conditions (chromosomal or monogenic ones) are frequently associated with CHD or CMP and can determine an overlapping phenotype [18].

One of the commonest examples in the field of complex systemic CMP is the group of RASopathies known for its major findings related to CHDs as PVS, ASD, VSD, AVCD, CoAo, TOF but also for CMP (HCM, less frequently DCM) in addition to progressive arrhythmic events. On the other hand, an increasingly growing example of single gene defects that can cause isolated CMP with or without CHD is related to *MYH7* (OMIM # 160760), a sarcomeric gene, which generally causes CMP or tachyarrhythmias but lately more and more observed in ASD, VSD, Ebstein anomaly, pulmonary artery hypoplasia and CoAo associated or not to myocardial involvement [18,19,20,21,22,23,24,25,26,27,28,29,30,31,32,33,34,35,36,37,38,39,40,41,42,43,44,45,46,47,48,49,50,51,52,53,54,55,56,57,58,59,60,61,62,63,64,65,66,67,68,69,70,71,72,73,74,75,76,77,78,79,80,81,82,83,84,85,86,87,88,89,90,91,92,93,94,95,96,97,98,99,100,101,102,103,104,105,106,107,108,109,110,111,112,113,114,115,116,117,118,119,120,121,122,123,124,125,126,127,128,129,130,131,132,133,134,135,136,137,138,139,140,141,142,143,144,145,146,147,148,149,150,151,152,153,154,155,156,157,158,159,160,161,162,163,164,165,166,167,168,169,170,171,172,173,174,175,176,177,178,179,180,181,182,183,184,185,186,187,188,189,190,191,192,193,194,195,196,197,198,199,200,201,202,203,204,205,206,207,208,209,210,211,212,213,214,215,216,217,218,219,220,221,222,223,224,225,226,227,228,229,230,231,232,233,234,235]. Moreover, Basu et al., 2014 has described BAV in a family with LVNC.

All the previous examples recommend major attention when observing CHDs, especially those with a complex or familial background, in order to avoid misdiagnosis of potential progressive myocardial and/or arrhythmic events.

## 5. Conclusions

Complex multisystemic pediatric CMP is a highly heterogeneous group of disorders. Standardized protocols that help clinicians in the diagnostic workup of these conditions are scarce in studies. In fact, complex CMP represents a rarity within a rarity.

**Figure 11:**
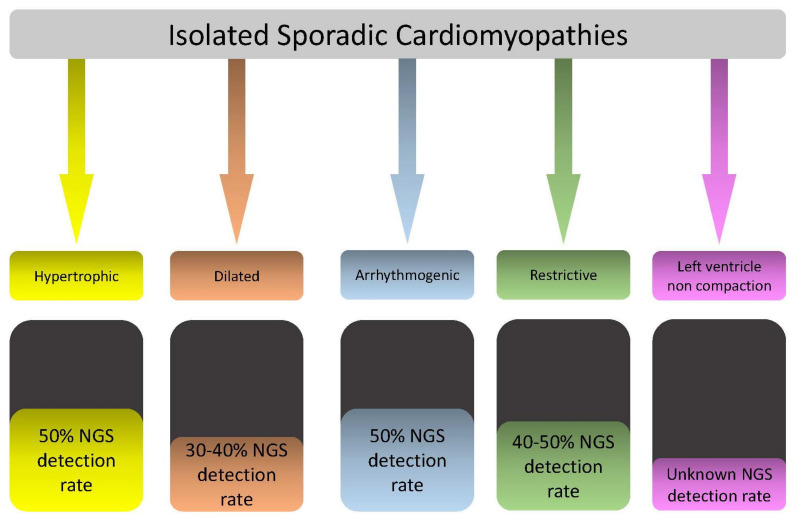
NGS detection rate in isolated CMPs. Abbreviations: CMPs, cardiomyopathies; NGS, next-generation sequencing.

However, for the majority of these rare disorders, we observed specific “red flags” associated with CMP that can be the focus to reach an appropriate diagnosis. These aspects can be gathered and processed along with the clinical workup that starts from history down to detailed investigations. In other words, the diagnostic process of complex CMP in children needs knowledge in cardiology, pediatrics, metabolic, radiology, and genetics (both clinical and molecular) (Figure 1 and Figure 11). Physicians who look after CMPs in children can face the most bizarre scenarios. It is rather an “artisan” work that needs personalized process. In large cohorts of systemic CMP, there may be a list of more than 30 to 40 different disorders that can not be repeated in two different patients over 20 years of cohort data gathering (personal observation, Baban 2021). The diagnostic process of CMP in children is rather a personalized medicine approach that does not need “anarchy” but a step-by-step method in order to deliver personalized management. Each data can represent a milestone within the diagnostic process of patients with complex multisystemic CMP. For example, starting from family history, consanguinity can be a red flag for AR conditions; specific clinical signs/symptoms (e.g., skin changes with wooly hair and PPK is a sign in CS), recurrent infections with neutropenia in a male is a sign of BTHS, but the same sign of recurrent infection in a dysmorphic infant with skin hypopigmentation and the absent corpus callosum is a sign of Vici syndrome. These “alarm bells” can quickly direct the clinicians towards specific disorders and set up a personalized path.

This approach can be extremely important for a rapid diagnostic and prognostic/therapeutic assessment. Time is often the worst enemy in the management of complex CMP.

## 6. Limitations

Our attempt to provide an algorithm based on specific “red flags” in the diagnostic process of CMP in children with complex conditions does not lack limitations. The major limitation is the extensive heterogeneity of the field. Moreover, it is well-known that some genetically determined conditions, particularly those with AD inheritance, are characterized by incomplete penetrance and phenotypic variability. The other difficulty in pediatric group is age-related penetrance and ethical issues when offering molecular testing to non-manifested children. These two genetic characteristics can make the diagnostic pathway difficult or slow it down. Moreover, genetic investigations are major, with a limited detection rate (Figure 11). The main focus of this paper is to observe children with CMP in a serial and global manner and not in a sectorial manner (only cardiac or static evaluation). In order to reach an appropriate diagnosis, family members genotyping and phenotyping are essential processes.
